# Artificial intelligence for the science of evidence synthesis: how good are AI-powered tools for automatic literature screening?

**DOI:** 10.1186/s12874-025-02644-9

**Published:** 2025-08-25

**Authors:** Minghao Ruan, Junhao Fan, Mingkai Liu, Zhengfeng Meng, Xiaohai Zhang, Chengjing Zhang

**Affiliations:** 1 The Third Department of Hepatic Surgery, Eastern Hepatobiliary Surgery Hospital, the Naval Medical University, Shanghai, China; 2 Department of Hepatic Surgery, Eastern Hepatobiliary Surgery Hospital, the Naval Medical University, Shanghai, China; 3 Proof of Concept Center, Eastern Hepatobiliary Surgery Hospital, the Naval Medical University, Shanghai, China; 4 School of Medicine, Shanghai Jiaotong University, Shanghai, China; 5 Health Management Center, Eastern Hepatobiliary Surgery Hospital, the Naval Medical University, Shanghai, China; 6 Department of Nutrition, Eastern Hepatobiliary Surgery Hospital, the Naval Medical University, Shanghai, China

**Keywords:** Artificial intelligence, Literature screening, Large language models, ChatGPT, Claude, Gemini, Deepseek, Robotsearch

## Abstract

**Background:**

Literature screening constitutes a critical component in evidence synthesis; however, it typically requires substantial time and human resources. Artificial intelligence (AI) has shown promise in this field, yet the accuracy and effectiveness of AI tools for literature screening remain uncertain. This study aims to evaluate the performance of several existing AI-powered automated tools for literature screening.

**Methods:**

This diagnostic accuracy study employed a cohort to evaluate the performance of five AI tools—ChatGPT 4.0, Claude 3.5, Gemini 1.5, DeepSeek-V3, and RobotSearch—in literature screening. We selected a random sample of 1,000 publications from a well-established literature cohort, with 500 as randomized controlled trials (RCTs) group and 500 as others group. Diagnostic accuracy was measured using several metrics, including the false negative fraction (FNF), time used for screening, false positive fraction (FPF), and the redundancy number needed to screen.

**Results:**

We reported the FNF for the RCTs group and the FPF for the others group. In the RCTs group, RobotSearch exhibited the lowest FNF at 6.4% (95% CI: 4.6% to 8.9%), whereas Gemini exhibited the highest at 13.0% (95% CI: 10.3% to 16.3%). In the others group, the FPF of the four large language models ranged from 2.8% (95% CI: 1.7% to 4.7%) to 3.8% (95% CI: 2.4% to 5.9%), both of which were significantly lower than RobotSearch's rate of 22.2% (95% CI: 18.8% to 26.1%). In terms of screening efficiency, the mean time used for screening per article was 1.3 s for ChatGPT, 6.0 s for Claude, 1.2 s for Gemini, and 2.6 s for DeepSeek.

**Conclusions:**

The AI tools assessed in this study demonstrated commendable performance in literature screening; however, they are not yet suitable as standalone solutions. These tools can serve as effective auxiliary aids, and a hybrid approach that integrates human expertise with AI may enhance both the efficiency and accuracy of the literature screening process.

**Graphical Abstract:**

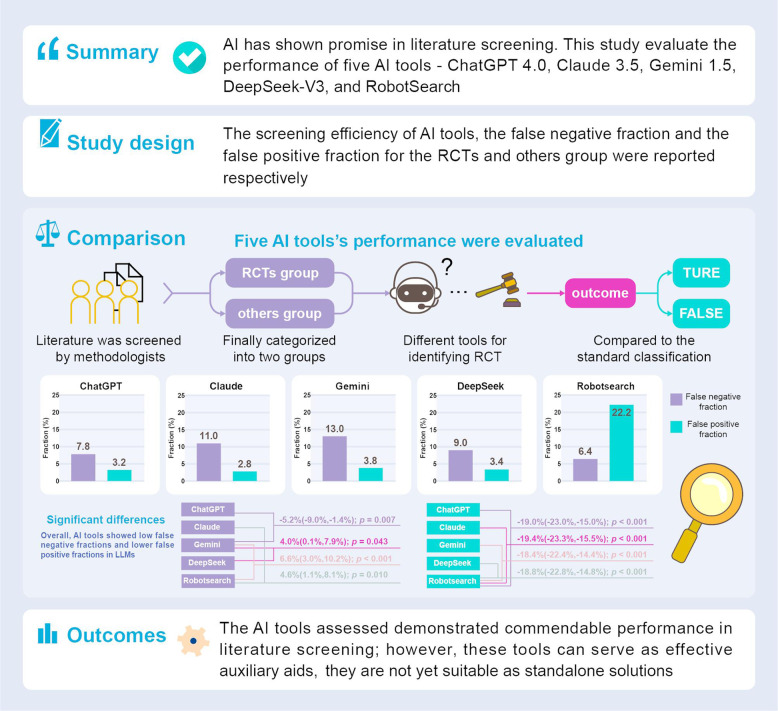

**Supplementary Information:**

The online version contains supplementary material available at 10.1186/s12874-025-02644-9.

## Introduction

Evidence synthesis, often referred to as research synthesis, is an essential methodology for aggregating evidence from existing studies to inform healthcare decision-making [1]. Due to its inherent characteristics, the primary objective of evidence synthesis is to address disagreements and delineate what is known and unknown regarding a specific topic [2]. As its value has gained increasing recognition, evidence synthesis has emerged as a fundamental component of the contemporary medical paradigm, producing evidences through these syntheses and supporting clinical guidelines and healthcare policies development [3].

In evidence synthesis practice, standardized procedures are crucial in safeguarding against potential biases (e.g., publication bias) and errors (e.g., human error), thereby preventing adverse effects on the validity of the summarized evidence [4]. A well-conducted evidence synthesis requires meticulous attention to detail at each step, making the process often time- and labor-intensive [5–7]. Generally, high-quality systematic reviews typically take 0.5 to 2 years to complete [8], raising concerns about efficiency in real-world scenarios where rapid and decisive healthcare decisions are often needed, especially during emergencies.

Literature screening is the most time-consuming process in evidence synthesis practice, particularly in the medical field [9–11], which involves two standardized steps: the initial screening of titles and abstracts, followed by reviewing full texts. Each step requires two researchers or groups working independently on the same task (i.e., double screening) [12, 13]. The first step aims to eliminate records that are clearly outside the scope of the specific topic, thereby reducing the workload for the full-text screening [14]. When researchers specify the study type (e.g., randomized controlled trials, RCTs) relevant to a particular topic, screening titles and abstracts becomes more manageable, as this information is usually included [10]. This facilitates the development of artificial intelligence for the screening process, thereby alleviating human workload and enhancing efficiency [15–17].

There are currently two types of AI-powered tools for literature screening: semi-automatic (e.g., Rayyan) and fully automatic AI-powered tools (e.g., RobotSearch) [10, 16–18]. Semi-automatic tools estimate and provide the probability of inclusion or exclusion for each literature, with final decisions made by human reviewers. In contrast, fully automatic tools complete the screening process and make decisions based entirely on algorithms, without human involvement [19–21]. While fully automatic tools are anticipated to be more efficient and seem to address the efficiency issue in evidence synthesis, their accuracy remains a key concern—specifically, whether the results produced are reliable. To address this question, we compared the performance of current fully automatic AI-powered tools. Our findings offer empirical evidence regarding the feasibility of utilizing AI-powered tools for literature screening.

## Methods

### Study design

This study is a diagnostic accuracy study with a cohort, where the population refers to the body of literature being screened. Briefly, a cohort of literature was established and reviewed by human reviewers following standardized procedures. The literature was then categorized into two groups: those are RCTs or others for equal size using simple random sampling. Using these RCTs and other samples, the diagnostic accuracy of AI-powered tools was evaluated and compared. This study focused specifically on the classification of RCTs versus others. An RCT was defined as a study in which participants were explicitly randomized into two or more groups, each receiving different interventions, as determined from the full text of the study [22]. The study was conducted in accordance with the STARD (Standards for Reporting Diagnostic Accuracy Studies) guidelines, where applicable [23].

### Literature cohort

The literature cohort for this study comprises 8,394 retractions sourced from the Retraction Watch database, up to April 26, 2023. Two experienced clinical epidemiology methodologists independently screened the exported records, following standard procedures. The initial screening involved a review of titles and abstracts, with only those records excluded by both reviewers being discarded. In the second stage, the remaining records—categorized as “definite”, “maybe”, or “controversial”—were assessed using full texts. Any discrepancies encountered during the full-text screening were resolved through discussion with a third senior methodologist. The Rayyan application (https://www.rayyan.ai/) was utilized for the literature screening, without employing the AI ranking system.

Following the literature screening, 779 retractions were identified as RCTs, while 7,595 were classified as not. To balance the sample sizes, a random sample of 500 articles was drawn from each group.

### AI-powered tools for literature screen

The current study evaluated the performance of the following five AI-powered tools: RobotSearch, ChatGPT 4.0, Claude 3.5, Gemini 1.5, and DeepSeek-V3. RobotSearch is an automatic literature classification tool specifically designed for identifying RCTs. It employs machine learning algorithms trained on a large dataset of RCTs from the Cochrane Crowd. This tool, developed by Marshall et al., is freely available to the public [24]. In contrast, ChatGPT 4.0, Claude 3.5, Gemini 1.5, and DeepSeek-V3 are large language models (LLMs) not specifically designed for RCT classification. However, these LLMs have demonstrated promising performance in general text classification tasks [10, 16, 25].

### Prompt engineering approach

The prompt engineering process consisted of three key steps. First, primary prompts were carefully developed and refined for literature screening task, with the assistance of LLMs to improve the initial designs. For example, a prompt such as “Please design the best prompt for me based on this prompt: …” was used in this step [26]. Second, iterative testing was conducted to further optimize the prompts. Finally, the refined prompts were applied to ChatGPT, Claude, Gemini, and DeepSeek to perform the literature screening tasks. See details in Table 1.

**Table 1:** Prompts for literature screening

Prompt
prompt = (
"Determine whether the provided literature (title, abstract) represents a randomized controlled trial (RCT).\n"
f"Please make a judgment based on the following paper title and abstract:\nTitle: {title} \nAbstract: {abstract}\nn"
"1. Review the title, abstract, and content of the PDF document.\n"
"2. Determine if the study involves random assignment of participants to different interventions.\n"
"3. Consider key indicators of RCTs such as \"randomized\", \"controlled\", \"trial\", \"random allocation\", and \"random assignment\".\n"
"4. If the study explicitly states it is an RCT or meets the criteria of an RCT, classify it as \"yes\".\n"
"5. If the study does not meet the criteria or lacks sufficient information to determine, classify it as \"no\".\n"
"output_format:\n"
"{\n"
"\"result\": \"yes\"or \"no\"\n"
"}\n"
"Output the json data only, with no additional text.")

### Outcomes

For automated literature screening tools, we assume that the most practically valuable tool should possess two key properties: (1) a near-zero false negative fraction (FNF), defined as the proportion of RCTs that are incorrectly excluded; and (2) a screening speed significantly faster than that of human reviewers. Accordingly, the primary outcome of this study is the false negative fraction of the tools. The secondary outcomes include the total time required for screening both the RCTs and others groups and the redundancy number needed to screen (RNNS). The RNNS is defined as the number of studies in the RCTs group that must still be manually reviewed after the automated screening process, specifically those incorrectly retained by the tool (false positives).

### Statistical analysis

We compared the false negative fraction (FNF) and its 95% confidence interval (CI) for each tool in the RCTs group and the false positive fraction (FPF) and its 95% CI in the others group. A lower FNF indicates a reduced likelihood of excluding studies that should be included, while a lower FPF reflects a reduced likelihood of including studies that should be excluded (or fewer studies requiring further manual screening). We calculated the Positive Likelihood Ratio (PLR = Sensitivity/(1 − Specificity)) to evaluate the diagnostic performance of each AI tool. The Youden’s Index (J = Sensitivity + Specificity − 1) provided additional insight into the overall test performance, allowing us to compare the effectiveness of different screening tools. The area under the curve (AUC) was not estimated because, in the context of literature screening for evidence synthesis, minimizing the false negative fraction (1 − sensitivity) is prioritized over minimizing the false positive fraction (1 − specificity). The percentage differences between the AI tools and the time required for screening were also calculated, with a significance level of α = 0.05. Risk Difference (RD = Risk in Exposed Group—Risk in Unexposed Group) and Risk Ratio (RR = Risk in Exposed Group/Risk in Unexposed Group) calculations complemented our analysis. The Number Needed to Screen (NNS), calculated as 1/J, offers a practical metric for understanding the efficiency of each screening tool. All statistical analyses were performed using Stata SE 16.0 (StataCorp LLC, College Station, TX).

## Results

Table 2 outlines the baseline characteristics of the included studies. Of these, 500 (50.0%) were RCTs, while the remaining 500 (50.0%) were not RCTs. Approximately one-third of the trials (36.0%) were published after 2019.

**Table 2:** Basic characteristics of trials

Basic characteristics	No. of studies (*n* = 1000)
Type of design	
RCTs	500(50.0%)
Others	500(50.0%)
Year of retraction	
Before 2010	364 (36.4%)
2011—2019	487 (48.7%)
After 2019	149 (14.9%)
Year of publication	
Before 2010	93 (9.3%)
2011—2019	547 (54.7%)
After 2019	360 (36.0%)

### Performance of automatic screen tools

#### False negative fraction

**Fig. 1:**
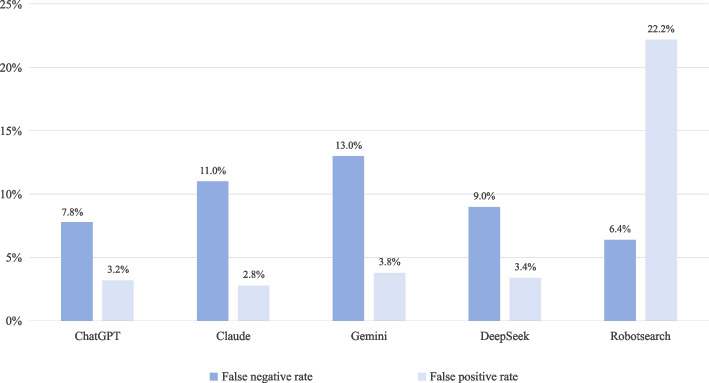
Performance of the AI tools for literature screening

Within the RCTs group, Robotsearch demonstrated the lowest FNF at 6.4% (95% CI: 4.6%, 8.9%) among the evaluated AI tools. In contrast, Gemini exhibited the highest FNF at 13.0% (95% CI: 10.3%, 16.3%). The FNF of the other AI tools are presented in Fig. 1.

**Fig. 2:**
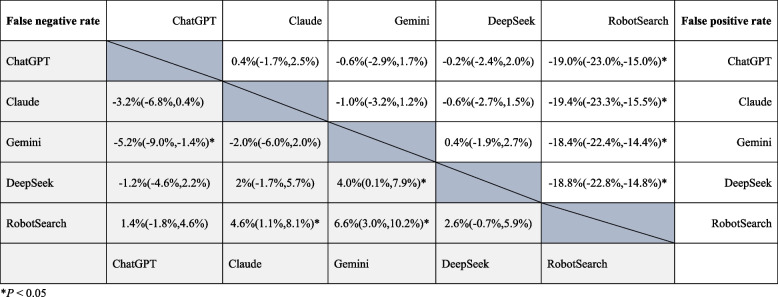
Percentage difference in the performance of the AI tools

Significant differences in FNF were observed between Gemini and the other AI tools, with percentage differences of −5.2% (ChatGPT vs. Gemini, 95% CI: −9.0%, −1.4%, *P* = 0.007), 4.0% (Gemini vs. Deepseek, 95% CI: 0.1%, 7.9%, *P* = 0.043), and 6.6% (Gemini vs. Robotsearch, 95% CI: 3.0%, 10.2%, *P* < 0.001). Additionally, a significant difference in FNF was observed between Claude and Robotsearch, with a percentage difference of 4.6% (95% CI: 1.1%, 8.1%, *P* = 0.010). No significant differences in FNF were found among ChatGPT, Claude, and Deepseek (see Fig. 2).

### Time used for screening

The time required for literature screening was recorded using the automatic timers of the LLMs. The total screening times for the RCTs and others groups were 22.0 min for ChatGPT, 100.0 min for Claude, 20.0 min for Gemini, and 43.5 min for Deepseek. On average, the time spent per article was 1.3 s for ChatGPT, 6.0 s for Claude, 1.2 s for Gemini, and 2.6 s for Deepseek.

### False positive fraction and the redundancy numbers required for screening

In the others group, the FPF for literature screening using LLMs were all below 4.0%. Gemini recorded the lowest FPF at 2.8% (95% CI: 1.7%, 4.7%), whereas Robotsearch exhibited the highest FPF, reaching 22.2% (95% CI: 18.8%, 26.1%) (see Fig. 1). The redundancy numbers required for screening with LLMs such as ChatGPT, Claude, Gemini, and DeepSeek were 16, 14, 19, and 17, respectively. In contrast, the redundancy number for Robotsearch was significantly higher at 111.

Robotsearch exhibited significantly higher FPF compared to the other tools. Significant differences in FPF were observed between Robotsearch and the four LLMs, with percentage differences of −19.0% (ChatGPT vs. Robotsearch, 95% CI: −23.0%, −15.0%, *P* < 0.001), −19.4% (Claude vs. Robotsearch, 95% CI: −23.3%, −15.5%, P < 0.001), −18.4% (Gemini vs. Robotsearch, 95% CI: −22.4%, −14.4%, *P* < 0.001), and −18.8% (Deepseek vs. Robotsearch, 95% CI: −22.8%, −14.8%, *P* < 0.001). However, no significant differences in FPF were observed among the four LLMs (ChatGPT, Claude, Gemini, and Deepseek) (see Fig. 2).

### Diagnostic performance evaluation

A comprehensive analysis revealed that ChatGPT demonstrated superior performance in literature screening, with a Positive Likelihood Ratio (PLR) of 28.812, Youden’s Index of 0.89, Number Needed to Screen (NNS) of 1.123, and Risk Difference (RD) and Risk Ratio (RR) of 0.89 and 28.813, respectively, revealing its exceptional screening capabilities. In contrast, Robotsearch showed significant limitations, with a PLR of 4.216, Youden’s Index of 0.714, NNS of 1.4, and substantially lower RD and RR values. Claude and Deepseek closely followed ChatGPT, while Gemini showed comparatively lower performance. Notably, all four large language models significantly outperformed Robotsearch in terms of FNF and FPF (see Table S1).

## Discussion

In this investigation, we assessed the performance of five automated literature screening tools: ChatGPT, Claude, Gemini, DeepSeek, and Robotsearch.

Regarding screening efficiency, ChatGPT, Gemini, and DeepSeek demonstrated a mean processing time of less than 3 s per article, whereas Claude required 6 s, which was notably longer than the other LLMs. Meanwhile, as demonstrated in prior studies [27], these tools also exhibit considerable potential for optimizing screening process efficiency. For instance, Claude processed each article in 6 s, DeepSeek in approximately 2.6 s, and both ChatGPT and Gemini in under 2 s. In contrast, human screening of the same dataset took approximately two weeks. Thus, AI tools are helpful for literature screening, but they cannot yet work independently without human supervision. We plan to investigate various strategies to enhance screening performance in the future, including the integration of human and AI-driven screening methods.

Among the evaluated AI tools, Robotsearch exhibited the lowest FNF at 6.4%, while Gemini had the highest at 13.0%. In the others group, the FPF for the four LLMs ranged from 2.8% to 3.8%, significantly lower than that of Robotsearch. Minimizing the FNF is crucial for an effective automated literature screening model, as it significantly reduces the human screening workload. Through this research, the AI tools demonstrated comparatively limited FNF in literature screening, outperforming results reported in some prior research [9, 16, 28]. Nevertheless, their performance remains inferior to that of humans [13, 16]. To optimize automated literature screening tools, it is crucial to minimize the risk of incorrectly excluding relevant studies; therefore, the ideal FNF should approach zero. Compared to previous studies, ChatGPT in our study achieve the relatively low FNF [16, 29]. In contrast, both Claude and Gemini exceeded 10.0%, with Gemini notably reaching a FNF of 13.0%, significantly higher than the other LLMs. This discrepancy may be attributed to Gemini's limited capacity to process complex medical texts [30]. These findings indicate that the literature screening capabilities of AI tools require further enhancement..

Our research also observed that LLMs achieve lower FPF compared to traditional RCT automated literature screening tools, such as Robotsearch. Consistent with previous research, the four LLMs evaluated in this study demonstrated consistently low FPF (all below 4%), with fewer than 20 articles requiring human review [10, 16]. These findings highlight the potential of LLMs to substantially alleviate the manual screening efforts. In contrast to traditional tools like Robotsearch, LLMs exhibited superior performance in reducing FPF. This may due to the limitations of traditional tools, which often segment text into fixed portions, potentially hindering their ability to capture contextual nuances, particularly in longer text [25]. LLMs, by leveraging their capabilities to capture contextual information and advanced semantic understanding [17], can more accurately identify relevant literature and enhance both the efficiency and accuracy of the screening process.

The diagnostic performance evaluation revealed the different literature screening capabilities of LLMs. The Youden’s Index, which comprehensively captures both sensitivity and specificity, demonstrated that ChatGPT (0.89) exhibited the most balanced performance, indicating excellent ability in literature screening. The Number Needed to Screen (NNS) further substantiated these findings, with ChatGPT requiring the least manual re-screening (1.123) compared to Robotsearch (1.4), suggesting enhanced screening efficiency. Risk Difference (RD) and Risk Ratio (RR) values reinforced these observations, with ChatGPT and Claude showing the most reliable screening outcomes. Therefore, the LLMs demonstrated substantial potential for optimizing literature screening processes compared to Robotsearch.

Significantly, our research found that ChatGPT exhibited superior literature screening capabilities. Recently, a large language model named DeepSeek has gained significant attention due to the exceptional performance of its upgraded V3 version. After our analysis DeepSeek-V3 for literature screening was assessed and found that its performance was comparable to that of ChatGPT. Therefore, both ChatGPT and DeepSeek can be regarded as robust tools for literature screening. However, further testing with larger and more diverse datasets is necessary to validate these findings.

To the best of our knowledge, this study represents the first attempt to employ multiple LLMs for literature screening. The enhanced data processing capabilities of LLMs demonstrate significant potential in this domain, offering several notable advantages. For instance, LLMs can rapidly process extensive volumes of literature, facilitating large-scale research initiatives while substantially reducing the time required for screening. Moreover, they can mitigate errors arising from human fatigue and subjective biases, thereby enhancing the objectivity of the screening process. Additionally, LLMs contribute to cost reduction in research and improve applicability by continuously learning and adapting to evolving research trends. Despite these advantages, LLMs currently serve as supplementary tools for human literature screening and require further refinement. Their integration with human expertise and ongoing model optimization is essential. By implementing these strategies, the utility of AI-driven tools in literature screening can be fully realized, maximizing their value in academic and research contexts.

Several limitations of this study should be acknowledged. First, the literature cohort from retraction database utilized in this research is current only as of April 26, 2023, and does not include databases related to other disease types, which may influence the outcomes of the literature screening. Second, due to the inherent variability in outputs generated by LLMs, this study did not assess the consistency of the same prompt across multiple iterations. Lastly, the literature screening approach employed here predominantly targets RCTs and does not encompass other types of studies. While RCTs often contain more explicit keyword information, the effectiveness of this screening methodology for other literature types remains to be validated in future research.

## Conclusion

In this study, AI tools demonstrated commendable performance in literature screening, particularly through the low FPF observed among the four LLMs. Notably, both LLMs completed the screening process in under six seconds per article, underscoring their potential to significantly reduce the time and effort required for screening tasks. These findings suggest that AI tools hold considerable promise for literature screening applications. However, their higher FNF compared to human screeners indicate that they are not yet suitable as standalone solutions. Instead, AI tools may function effectively as auxiliary aids, and adopting a hybrid human-AI approach could optimize both the efficiency and accuracy of the screening process.

## Supplementary Information

Supplementary Material 1: Table S1. Diagnostic Performance Evaluation of LLMs in Literature Screening.

Supplementary Material 2.

## Data Availability

No datasets were generated or analysed during the current study.

## Abbreviations

RCTs: Randomized controlled trials
AI: Artificial intelligence
LLMs: Large language models
FNF: False negative fraction
RNNS: Redundancy number needed to screen
CI: Confidence interval
FPF: False positive fraction
AUC: Area under the curve
PLR: Positive Likelihood Ratio
NNS: Number Needed to Screen
RD: Risk Difference
RR: Risk Ratio
