# Supplementary material for: Artificial intelligence for the science of evidence synthesis: how good are AI-powered tools for automatic literature screening?
Source: BMC Med Res Methodol. 2025 Aug 25;25:199. doi: 10.1186/s12874-025-02644-9 (PMC12376440; doi:10.1186/s12874-025-02644-9)
Supplement: Supplementary file 1 — Supplementary Material 1: Table S1. Diagnostic Performance Evaluation of LLMs in Literature Screening. [file 12874_2025_2644_MOESM1_ESM.docx]

| Table S1. Diagnostic Performance Evaluation of LLMs in Literature Screening | | | | | | | |  |
| --- | --- | --- | --- | --- | --- | --- | --- | --- |
| Metrics | Sensitivity | Specificity | PLR | Youden’s Index | NNS | RD | RR | |
| ChatGPT | 0.922 | 0.968 | 28.812 | 0.89 | 1.123 | 0.89 | 28.813 | |
| Claude | 0.89 | 0.972 | 31.786 | 0.862 | 1.16 | 0.862 | 31.786 | |
| Gemini | 0.87 | 0.962 | 22.895 | 0.832 | 1.201 | 0.832 | 22.895 | |
| DeepSeek | 0.91 | 0.966 | 26.765 | 0.876 | 1.141 | 0.876 | 26.765 | |
| RobotSearch | 0.936 | 0.778 | 4.216 | 0.714 | 1.4 | 0.714 | 4.216 | |
